# Plasma Lipocalin 2 in Alzheimer’s disease: potential utility in the differential diagnosis and relationship with other biomarkers

**DOI:** 10.1186/s13195-021-00955-9

**Published:** 2022-01-13

**Authors:** Peter Hermann, Anna Villar-Piqué, Matthias Schmitz, Christian Schmidt, Daniela Varges, Stefan Goebel, Timothy Bunck, Hanna Lindemann, Carla Bogner, Isabel Santana, Inês Baldeiras, Joachim Riggert, Inga Zerr, Franc Llorens

**Affiliations:** 1 Department of Neurology, Clinical Dementia Center and National Reference Center for CJD Surveillance, University Medical Center Göttingen, Robert-Koch Street 40, 37075 Göttingen, Germany; 2 Network Center for Biomedical Research in Neurodegenerative Diseases, (CIBERNED), Institute Carlos III, L’Hospitalet del Llobregat, Spain; 3 Bellvitge Biomedical Research Institute (IDIBELL), L’Hospitalet de Llobregat, Spain; 4 German Center for Neurodegenerative Diseases (DZNE), Göttingen, Germany; 5 Neurology Department, CHUC - Centro Hospitalar e Universitário de Coimbra, CNC- Center for Neuroscience and Cell Biology, University of Coimbra, Coimbra, Portugal; 6 Department of Transfusion Medicine, University Medical Center, Göttingen, Germany

**Keywords:** Dementia, Alzheimer’s disease, Biomarker, Plasma, Lipocalin 2, Neutrophil gelatinase-associated Lipocalin

## Abstract

**Background:**

Lipocalin-2 is a glycoprotein that is involved in various physiological and pathophysiological processes. In the brain, it is expressed in response to vascular and other brain injury, as well as in Alzheimer’s disease in reactive microglia and astrocytes. Plasma Lipocalin-2 has been proposed as a biomarker for Alzheimer’s disease but available data is scarce and inconsistent. Thus, we evaluated plasma Lipocalin-2 in the context of Alzheimer’s disease, differential diagnoses, other biomarkers, and clinical data.

**Methods:**

For this two-center case-control study, we analyzed Lipocalin-2 concentrations in plasma samples from a cohort of *n* = 407 individuals. The diagnostic groups comprised Alzheimer’s disease (*n* = 74), vascular dementia (*n* = 28), other important differential diagnoses (*n* = 221), and healthy controls (*n* = 84). Main results were validated in an independent cohort with patients with Alzheimer’s disease (*n* = 19), mild cognitive impairment (*n* = 27), and healthy individuals (*n* = 28).

**Results:**

Plasma Lipocalin-2 was significantly lower in Alzheimer’s disease compared to healthy controls (*p* < 0.001) and all other groups (*p* < 0.01) except for mixed dementia (vascular and Alzheimer’s pathologic changes). Areas under the curve from receiver operation characteristics for the discrimination of Alzheimer’s disease and healthy controls were 0.783 (95%CI: 0.712–0.855) in the study cohort and 0.766 (95%CI: 0.627–0.905) in the validation cohort. The area under the curve for Alzheimer’s disease versus vascular dementia was 0.778 (95%CI: 0.667–0.890) in the study cohort. In Alzheimer’s disease patients, plasma Lipocalin2 did not show significant correlation with cerebrospinal fluid biomarkers of neurodegeneration and AD-related pathology (total-tau, phosphorylated tau protein, and beta-amyloid 1-42), cognitive status (Mini Mental Status Examination scores), APOE genotype, or presence of white matter hyperintensities. Interestingly, Lipocalin 2 was lower in patients with rapid disease course compared to patients with non-rapidly progressive Alzheimer’s disease (*p* = 0.013).

**Conclusions:**

Plasma Lipocalin-2 has potential as a diagnostic biomarker for Alzheimer’s disease and seems to be independent from currently employed biomarkers.

**Supplementary Information:**

The online version contains supplementary material available at 10.1186/s13195-021-00955-9.

## Background

The ante-mortem definition of Alzheimer’s disease (AD) underwent a substantial evolution over the past 20 years. Cerebrospinal fluid (CSF) biomarkers reflecting AD-related pathological changes such as CSF phosphorylated Tau protein (p-tau) and Beta-amyloid 1-42 (Abeta42), as well as markers of neurodegeneration like total-Tau (t-tau), were identified, validated, and employed in established research criteria for the diagnosis of AD [1, 2]. Recently proposed criteria may even allow prodromal or preclinical diagnosis if evidence for AD-related Abeta- or tau-pathology is detected through CSF analyses or positron emission tomography (PET) [3]. The next step and current focus of biomarker research is the validation of minimal-invasive tests using blood plasma or serum [4]. Promising diagnostic and prognostic values of assays detecting elevated p-tau [5, 6], Abeta42 [7], and markers of neuro-axonal damage like Neurofilament light chain (NfL) [8] and t-tau [9] have been recently reported. Beyond this, biomarkers of other aspects of AD-pathology (e.g., neuro-inflammation or synaptic damage) are needed to improve diagnosis and to monitor specific therapeutic aims in clinical trials [10].

Lipocalin 2 (LCN2), also named Neutrophil Gelatinase-Associated Lipocalin, is a secreted glycoprotein, involved in innate immunity and brain iron homeostasis, and expressed in the brain in response to injury and inflammation [11]. Further, LCN2 mediates hippocampal damage in a model of vascular dementia (VaD) [12] and high CSF LCN2 levels were reported to be a promising diagnostic biomarker for VaD [13], whereas decreased levels of CSF LCN2 were found in patients with mild cognitive impairment (MCI) due to AD [14]. Peripheral LCN2 has been evaluated and established as a biomarker for kidney injury [15] but was also proposed as a potential blood-based biomarker for intestinal inflammation [16] and Alzheimer’s disease [17]. Regarding the latter, available data is scarce and inconsistent. Some previous studies reported unaltered [14, 18] or even slightly elevated plasma or serum LCN2 levels in patients with mild cognitive impairment (MCI) [19] and preclinical AD [20].

Here, we aimed to investigate plasma LCN2 in the context of the differential diagnoses of dementia and its utility as an independent biomarker by analyzing associations with biomarkers of AD-related pathology as well as with clinical data in AD patients. Further, we evaluated differences between AD patients with normal and rapid progressions to explore a potential prognostic utility.

## Methods

### Study design, participants, and data acquisition

For this retrospective two-center case-control study, we analyzed LCN2 levels in plasma samples from cases with AD, VaD, MCI, and important differential diagnoses, as well as in healthy controls (HC) from two independent cohorts (*n* = 481). Data and samples from cohort 1 (*n* = 407) were collected in the Clinical dementia center at the University Medical Center Göttingen (Germany) through prospective studies on AD, VaD, and Creutzfeldt-Jakob disease (CJD) Surveillance. Cases were selected on the base of availability of plasma samples, clinical information, and sufficient diagnostic characterization. HC were obtained from the Department of Transfusion Medicine. Cohort 2 (*n* = 74) was analyzed to validate the main findings from Cohort 1 in AD, MCI, and HC (caregivers, that accompanied patients) that were recruited at the Dementia Clinic of the Coimbra University Hospital (Portugal). Clinical and demographic information has been recorded during the diagnostic process through standardized questionnaires including third-party anamneses. White matter hyperintensities on MRI (FLAIR or T2 weighted images) in the AD group (cohort 1) were assessed using the age-related white matter changes (ARWMC) scale [21]. Global cognitive status was tested with the Mini Mental Status Examination (MMSE) [22]. All participants or their legal representatives gave written informed consent for analysis of their biological samples and publication of the data.

### Diagnostic criteria

Probable AD was diagnosed according to the National Institute on Aging - Alzheimer's Association workgroups (NIA-AA) criteria [1]. In addition, AD patients showed one or more positive biomarkers according to the A (amyloid-pathology)/ T (AD-related tau-pathology)/ N (neurodegeneration) system [23]. Stratification of AD cases in slowly progressive AD (spAD) and rapidly progressive AD (rpAD) was based on the rate of cognitive decline, indicating rpAD when MMSE scores declined more than 5 points per year [24]. Amnestic MCI (in cohort 1 and 2) was defined as the presence of mild cognitive deficits with memory impairment and unimpaired activities of daily living, matching the clinical NIA-AA criteria for diagnosis of MCI [25]. The MCI-AD group (cohort 1) included only amnestic MCI patients with at least on positive AD-related CSF biomarker, either reflecting Abeta-pathology (low Abeta 1-42, Abeta 1-42/1-40 ratio) or AD-related tau-pathology (elevated p-tau).

VaD diagnosis was based on guidelines from the Vascular Impairment of Cognition Classification Consensus Study and included a complete clinical work up showing no evidence for other than vascular pathology of the brain. Mild vascular cognitive impairment (MCI-VCI) was diagnosed when patients fulfilled criteria for VCI but had unimpaired activities of daily living [26]. CSF p-tau and Abeta 1-42 were considered to exclude concomitant AD pathology in vascular patients as far as possible. The mixed dementia (MD) group included patients according to clinical International Working Group (IWG-2) criteria [2] and also patients with VaD according to NINDS-AIREN criteria plus at least one AD-typical CSF biomarker (elevated phosphorylated-tau, low Abeta 1-42, or low Abeta 1-42/1-40 ratio). Sporadic CJD, dementia with Lewy bodies and Parkinson’s disease dementia (Lewy body diseases, LBD), and fronto-temporal dementia (FTD) were diagnosed according to clinical consensus criteria [27–29]. The HC group included healthy blood donors without evidence for CNS or clinically relevant peripheral diseases. Another control group (ND-Dem) included patients with neurological diseases and dementia of primarily non-neurodegenerative causes: cerebral vasculitis, normal-pressure hydrocephalus, Wilson’s disease, CNS neoplasia, encephalitis, and dementia due to alcohol abuse. Criteria for clinical diagnoses were assessed at the same patient visitation or within the same clinic stay. Concomitant CNS pathologies as well as severe non-CNS pathologies (neoplasia, autoimmune-diseases, clinically decompensated heart, lung, or kidney failure) were excluded in all diagnostic groups. The presence of chronic renal failure was evaluated through medical reports from the Göttingen university hospital or other institutions of treatment. This condition was not present in HC, MCI, and AD groups but not excluded in other dementia groups.

### Fluid biomarker measurement

Blood was collected in EDTA tubes randomly throughout the day and centrifuged at 1500×*g* at 4 °C for 10 min under same pre-analytical conditions. For quantification of plasma LCN2, a human LCN2/NGAL (Neutrophil Gelatinase-Associated Lipocalin) Quantikine Enzyme-linked Immunosorbent Assay (ELISA) Kit (R&D Systems, Inc. Minneapolis, MN) was used and manufacturer’s instructions were followed. Plasma samples were diluted 1:100. We calculated an inter-assay coefficient of variability of 6.98 and an intra-assay coefficient of variability of 6.03. Before application, we performed a small study on the influence of pre-analytical conditions, indicating that plasma LCN2 concentrations are stable after three transfers, three freeze-thaw cycles (room temperature/minus 80°C), and 8 days storage at 4°C (Additional file 1A). Only samples that matched the tested criteria were included for further analyses.

CSF t-tau, p-tau, Abeta 42, and Abeta 40 were quantified using ELISA kits from Fujirebio (Fujirebio, Ghent, Belgium). The established lab-specific cut-offs indicating pathological levels t-tau > 449 pg/ml, p-tau > 60 pg/ml, Abeta 1-42 < 450 pg/ml, and Abeta ratio [(abeta1-42/abeta1-40)*10)] <0.975 were applied as previously reported [30]. Test performers were blind to clinical information and clinical investigators vice versa.

### Statistical methods

In the pre-analytical study, differences were tested with repeated ANOVA followed by Bonferroni post hoc test. Comparison of biomarker levels among diagnostic groups was performed with linear regression models. Biomarker data were log-transformed; age and sex were included as covariates in all models. Multiple comparisons of means were performed with Tukey contrasts available in the multcomp R package [31]. Spearman rank order correlation was used to analyze associations between continuous biomarker levels. To assess the diagnostic accuracies, receiver operating characteristic (ROC) curve analyses were used to calculate areas under the curve (AUC). All statistical calculations and graphical representations were performed with GraphPad Prism 5 software, except linear regression models, which were computed in R. Statistical significance was considered at *p* < 0.05.

## Results

### Cohorts, groups, and demographic data

Cohort 1 included *n* = 407 cases with AD (*n* = 74), VaD (*n* = 28), MD (*n* = 7), other neurodegenerative dementia entities (CJD, *n* = 84; LBD, *n* = 45; FTD, *n* = 30), ND-Dem (*n* = 25), and HC (*n* = 84). In addition, two non-dementia groups with MCI (MCI-AD, *n* = 14; MCI-VCI, *n* = 16) were included. The AD group was furtherly characterized as spAD (*n* = 46) and rpAD (*n* = 28). Mean age ranged from 60.3 (ND-Dem) to 71.4 (VaD) over all groups and distribution of sexes was uneven (Table 1). Therefore, sex and age were included as potential confounding factors in all statistical models for group comparisons. Cohort 2 included patients with AD (*n* = 19), amnestic MCI (*n* = 27), and HC (*n* = 28).

**Table 1:** Demographic data and biomarker concentrations

	***n***	***Age (mean ± SD)***	***Sex (f/m)***	***MMSE (median, IQR)***	***CSF (mean*** ± ***SD)***			***Plasma (mean ± SD)***
					***t-tau (pg/mL)***	***p-tau (pg/mL)***	***Ab42 (pg/mL)***	***LCN2 (ng/mL)***
***Cohort 1***								
HC	84	64.0 ± 5.3	26/58	NA	NA	NA	NA	105.0 ± 53.7
ND-Dem	25	60.3 ± 15.2	16/9	NA	484 ± 402	NA	NA	78.9 ± 28.1
AD	74	67.6 ± 9.6	38/36	20.0 (9.0)^a^	622 ± 434	100.5 ± 58.3	489 ± 317	58.3 ± 28.0
spAD	48	67.4 ± 9.6	22/26	22.5 (8.3)	510 ± 312	85.3 ± 34.1	502 ± 266	63.3 ± 28.3
rpAD	26	67.8 ± 9.6	16/10	17.0 (10.5)	835 ± 540	125 ± 73.9	384 ± 176	49.1 ± 25.7
MCI-AD	14	68.7 ± 7.7	6/8	27.0 (3.0)	571 ± 322	88.3 ± 27.6	569 ± 292	103.1 ± 42.2
MD	7	70.3 ± 10.5	4/3	18.0 (12.5)^a^	406 ± 284	91.1 ± 27.6	491 ± 253	53.4 ± 23.9
VaD	28	71.4 ± 9.9	20/8	21 (10.0)^a^	333 ± 325	47.3 ± 17.8	754 ± 374	135.0 ± 120.3
VCI-MCI	16	69.5 ± 8.2	11/5	28.0 (2.3)	174 ± 62.0	40.7 ± 10.5	983 ± 195	90.1 ± 24.8
CJD	84	65.7 ± 11.8	51/33	NA	8859 ± 7463	57.9 ± 19.7	482 ± 286	102.3 ± 58.8
FTD	30	65.6 ± 11.4	17/13	20.5 (10.3)^a^	371 ± 401	59.8 ± 43.6	693 ± 293	83.2 ± 59.5
LBD	45	70.3 ± 9.7	17/28	21.0 (10.3)^a^	320 ± 210	46.9 ± 25.1	637 ± 287	82.2 ± 31.7
***Cohort 2***								
HC	28	72.1 ± 6.9^b^	19/9	NA	NA	NA	NA	97.6 ± 49.0
Amnestic MCI	27	67.7 ± 9.0	12/15	NA	NA	NA	NA	80.5 ± 21.8
AD	19	69.7 ± 6.0	8/11	NA	610 ± 314	65.0 ± 28.6	390 ± 108	65.1 ± 21.0

*HC* Healthy controls, *ND-Dem* Non-neurodegenerative neurological diseases with dementia syndrome, *AD* Alzheimer’s disease (dementia), *rpAD* Rapidly progressive Alzheimer’s disease, *spAD* Slowly progressive Alzheimer’s disease, *MCI-AD* Mild cognitive impairment with positive AD-related biomarkers, *MD* Mixed dementia (AD plus vascular), *VaD* Vascular dementia, *VCI-MCI* Mild vascular cognitive impairment, *CJD* Creutzfeldt-Jakob disease, *FTD* Fronto-temporal dementia, *LBD* Lewy body diseases (dementia with Lewy bodies and Parkinson’s disease dementia), *MMSE* Mini Mental Status Examination score, *SD* Standard error, *t-Tau* CSF Total tau protein, *p-tau* CSF Phosphorylated tau protein, *Abeta42* CSF beta-amyloid 1-42, *SD* Standard deviation, *IQR* Interquartile range

^a^Available MMSE scores (if different from total group size) in AD: *n* = 71, MD: *n* = 6, VaD: *n* = 23, FTD: *n* = 14; DLB: *n* = 34

^b^For 3 cases, age was not available

### Group comparisons and diagnostic accuracy of plasma LCN2

**Fig. 1:**
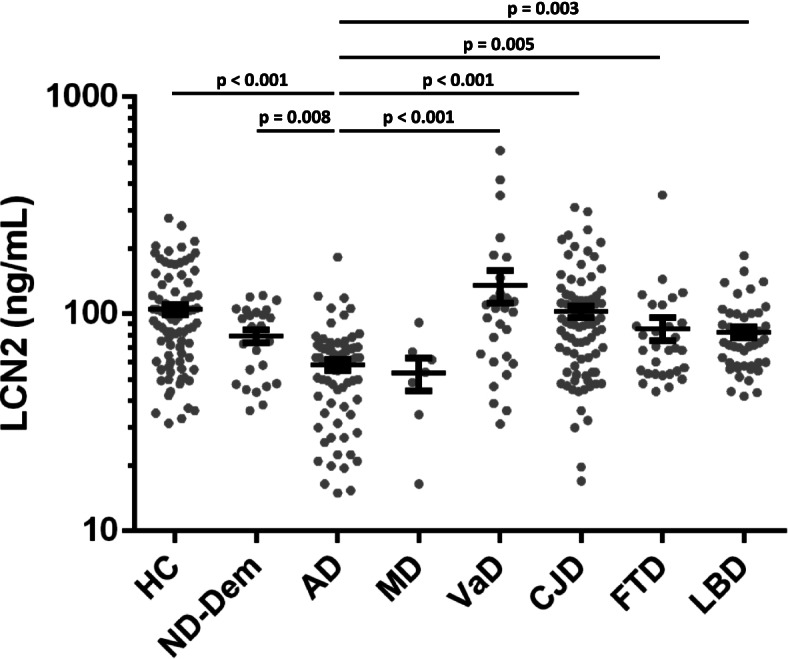
Plasma LCN2 in the differential diagnosis of dementia. Plasma LCN2 concentrations in diagnostic groups. Comparison of biomarker concentrations of diagnostic groups was performed with linear regression models; age and sex were included as covariates. Multiple comparisons were performed with Tukey contrasts. *P*-values are indicated above dot columns. Bars indicate mean and standard error of mean. MD group is presented for visualization purposes but excluded from data analyses due to the low number of cases. HC, healthy controls; ND-Dem, non-neurodegenerative neurological diseases with dementia syndrome; AD, Alzheimer’s disease; CJD, Creutzfeldt-Jakob disease; LBD, Lewy body diseases (dementia with Lewy bodies and Parkinson’s disease dementia); FTD, fronto-temporal dementia; and VaD, vascular dementia

The lowest plasma LCN2 concentrations in cohort 1 were observed in the AD group (mean: 58.3 ng/ml, SD ± 28.0). This was significantly lower than in HC (mean: 105.0 ng/ml, SD ± 53.7, *p* < 0.001), ND-Dem (mean: 78.9 ng/ml, SD ± 28.1, *p* = 0.008), VaD (mean: 135.0 ng/ml ± SD 120.3, *p* < 0.001), CJD (mean: 102.3 ng/ml, SD ± 58.8, *p* < 0.001), FTD (mean: 83.2 ng/ml, SD ± 59.5, *p* = 0.005), and LBD (mean: 82.2 ng/ml, SD ± 31.7, *p* = 0.008) (Table 1, Fig. 1). Detailed results from the statistical model are shown in Additional file 1B. The MD group (mean 53.4 ng/ml, SD ± 23.9) showed a LCN2 concentration similar to AD but was excluded from data analyses due to the low number of cases (*n* = 7). The highest mean concentrations of plasma LCN2 were observed in the VaD group (mean: 135.0 ng/ml, SD ± 120.3) but the differences versus other groups, as well as differences between other dementia groups and HC, were not statistically significant (Additional file 1B). We calculated AUCs for the discrimination of AD from each in cohort 1. Highest AUCs were observed for AD vs. HC (AUC = 0.783, 95% CI 0.712 to 0.855) and AD vs. VaD (AUC = 0.778, 95% CI 0.667 to 0.890). Lowest AUC was observed for AD vs. MD (AUC = 0.562, 95% CI 0.349 to 0.775) (Table 2).

**Table 2:** Diagnostic accuracy of plasma LCN2

Diagnostic groups	AUC (95% CI)	***P***-value
AD vs. HC	0.783 (0.712–0.855)	*p* < 0.001
AD vs. ND-Dem	0.694 (0.567–0.821)	*p* = 0.004
AD vs. MD	0.562 (0.349–0.775)	*p* = 0.591
AD vs. VaD	0.778 (0.667–0.890)	*p* < 0.001
AD vs. CJD	0.760 (0.685–0.835)	*p* < 0.001
AD vs. FTD	0.682 (0.571–0.793)	*p* = 0.004
AD vs. LBD	0.7221 (0.629–0.815)	*p* < 0.001

*HC* Healthy controls, *ND-Dem* Non-neurodegenerative neurological diseases with dementia syndrome, *AD* Alzheimer’s disease (dementia), *MD* Mixed dementia (AD plus vascular), *VaD* Vascular dementia, *VCI-MCI* Mild vascular cognitive impairment, *CJD* Creutzfeldt-Jakob disease, *FTD* Fronto-temporal dementia, *LBD* Lewy body diseases (dementia with Lewy bodies and Parkinson’s disease dementia), *AUC* Area under the curve, *CI* Confidence interval

**Fig. 2:**
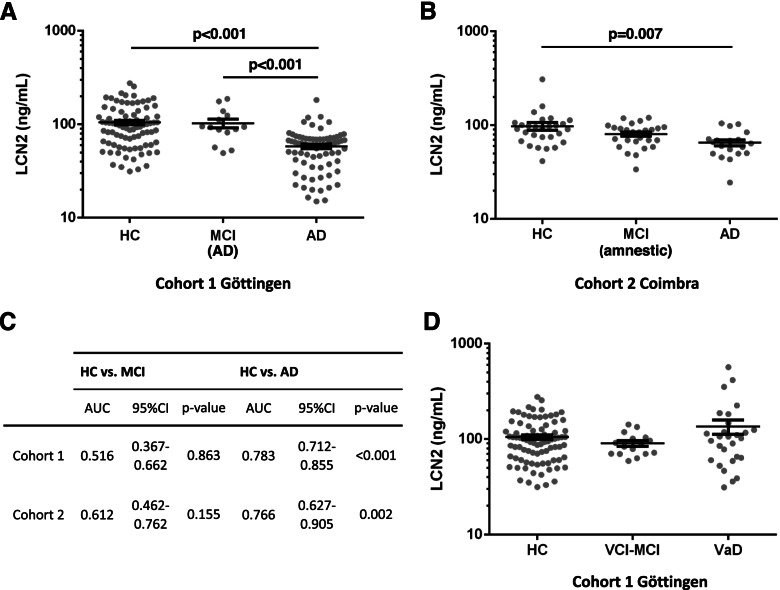
Plasma LCN2 in AD and MCI cases of cohorts 1 and 2. **A** Plasma LCN2 concentrations in HC, MCI-AD, and AD of cohort 1. **B** Plasma LCN2 concentrations in HC, amnestic MCI, and AD of cohort 2. **C** Areas under the curve (AUC) from receiver operating characteristic with 95% confidence intervals (95% CI) and *p*-values from assessment of diagnostic accuracy in cohort 1 and 2. **D** Plasma LCN2 concentrations in HC, MCI-VCI, and VaD of cohort 1. Comparison of biomarker concentrations of diagnostic groups was performed with linear regression models; age and sex were included as covariates. Multiple comparisons were performed with Tukey contrasts. *P*-values are indicated above dot columns when significant. Bars (**A**, **B**, **D**) indicate mean and standard error of mean. HC, healthy controls; MCI (AD), mild cognitive impairment with positive biomarkers for AD-related pathology; AD, Alzheimer’s disease; MCI (amnestic), mild cognitive impairment with disturbance of memory function; MCI-VCI, mild vascular cognitive impairment; VaD, vascular dementia or major vascular cognitive impairment

Additional comparison models included HC, AD, and prodromal AD (MCI-AD and amnestic MCI, respectively) in cohort 1 and cohort 2. Plasma LCN2 concentration was significantly lower in AD (mean: 58.3 ng/ml, SD ± 28.0) compared to both, HC (mean: 105.0 ng/ml, SD ± 53.7, *p* < 0.001) and MCI-AD (mean: 103.1 ng/ml, SD ± 42.2, *p* < 0.001) in cohort 1 (Fig. 2A), and compared to HC (mean: 97.6 ng/ml, SD ± 49.0, *p* = 0.007) in cohort 2 (Fig. 2B). In both cohorts, no significant differences between HC and MCI groups were detected. Detailed results from the statistical models are shown in Additional file 1C. The AUC of AD vs. HC in was (AUC = 0.766, 95% CI 0.672 to 0.905) in the validation cohort (Fig. 2C). The diagnostic accuracy of plasma LCN2 in the differentiation of HC vs. MCI-AD (AUC = 0.515, 95% CI 0.367 to 0.662, cohort 1) and HC vs. amnestic MCI (AUC = 0.612, 95% CI 0.462 to 0.762, cohort 2) was rather low, though (Fig. 2C). No significant differences could be observed in a corresponding model for patients with cerebrovascular pathology (only cohort 1) including HC, MCI-VCI, and VaD (Fig. 2D, Additional file 1C).

### Associations of plasma LCN2 with other biomarkers

**Fig. 3:**
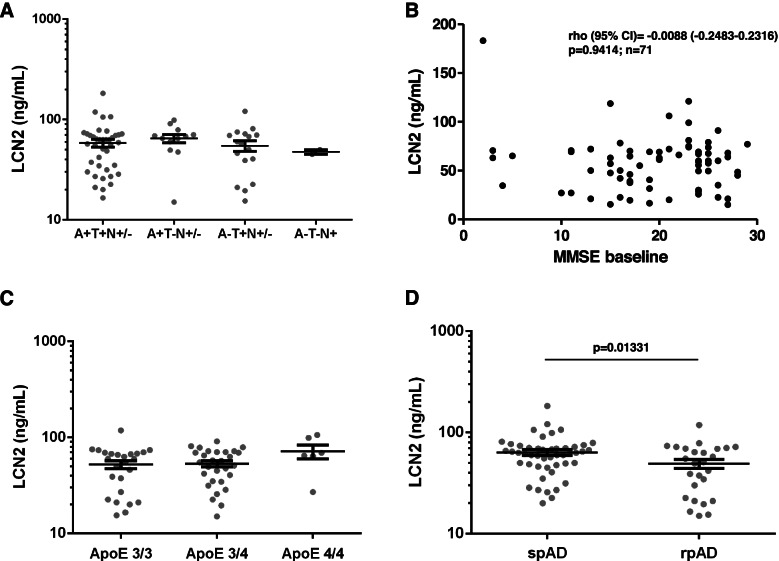
Disease stage, clinical subtypes, APOE genotype, and white matter hyperintensities in AD in cohort 1. **A** Plasma LCN2 concentrations in AD patients with different biomarker characteristics. Group comparisons were performed with linear regression models; age and sex were included as covariates. Multiple comparisons were performed with Tukey contrasts. No significant differences were found. A+/−, positive/negative for decreased CSF Abeta 1-42 or Abeta 1-42/1-40 ratio; T+/−, positive/negative for increased CSF p-tau; N+, positive marker of neurodegeneration, either elevated CSF t-tau or medial temporal lobe atrophy on MRI. **B** Scatter plot of the association between Mini Mental Status Examination (MMSE) scores and plasma lipocalin 2 (LCN2) concentrations. Spearman coefficients (cc) with 95% confidence interval (CI) and corresponding *p-*values are indicated. **C** Plasma LCN2 concentrations in different APOE genotypes in the AD group. Comparison of biomarker concentrations was performed with linear regression models; age and sex were included as covariates. Multiple comparisons were performed with Tukey contrasts. No significant differences were found. **D** Plasma LCN2 in rapidly progressive Alzheimer’s disease (rpAD) and slowly progressive Alzheimer’s disease (spAD). For comparison between the two groups, a linear regression model including age and sex as covariates was applied; the corresponding *p*-value is indicated. Bars (**B**, **C**, and **D**) indicate mean and standard error of mean

We explored potential associations of plasma LCN2 with AD-related CSF biomarkers (p-tau, Abeta) and CSF t-tau as a marker of neuro-axonal injury in AD cases from cohort 1. Employing Spearman non-parametric correlation, no statistically significant associations were found. The same analyses were performed in patients with CJD, LBDs, FTD, and VaD but no statistically significant correlations were observed either (Table 3). To explore associations with AD-related biomarkers further, we stratified AD patients according to the A/T/N system and obtained four major groups. Plasma LCN2 concentrations in patients with A+/T− (only Abeta positive, *n* = 12, mean: 64.9 ng/ml, SD ± 21.6), A-/T+ (only p-tau positive, *n* = 17, mean: 54.6 ng/ml, SD ± 27.3), A+/T+ (*n* = 39, mean: 58.2 ng/ml, SD ± 32.1), and A−/T−/N+ (only markers for neurodegeneration positive, *n* = 2, mean: 47.5 ng/ml , SD ± 3.5) showed no significant differences in the multi-comparison model (Fig. 3A, Additional file 1C). In addition, a multi-comparison model including only AD cases with A+/T+ biomarker profile was calculated and showed that LCN2 concentrations in the AD A+/T+ group were significantly lower compared to all other diagnostic groups (Additional file 1D). Regarding MCI-AD cases in cohort 1, ten of fourteen patients had increased CSF p-tau and decreased Abeta 1-42 (LCN2 mean 101.5 ng/ml); four patients had only increased p-tau (LCN mean: 106 ng/ml). Due to the small group sizes, further analyses were not performed.

**Table 3:** Correlations of plasma LCN2 and CSF biomarkers in dementia groups

	t-tau	p-tau	Abeta42	Abeta40
**AD**				
rho	−0.098	−0.112	0.182	0.070
95% CI	−0.332 to 0.147	−0.344 to 0.134	−0.406 to 0.062	−0.193 to 0.323
*p*-value	0.420	0.358	0.131	0.592
**VaD**				
rho	0.281	−0.141	−0.030	
95% CI	−0.114 to 0.600	−0.517 to 0.280	−0.438 to 0.389	n.a.
*p*-value	0.147	0.501	0.891	
**LBD**				
rho	0.078	−0.119	−0.296	
95% CI	−0.233 to 0.374	−0.444 to 0.233	−0.560 to 0.022	n.a.
*p*-value	0.617	0.495	0.060	
**CJD**				
rho	−0.028	−0.071	0.105	
95% CI	−0.247 to 0.103	−0.372 to 0.243	−0.168 to 0.362	n.a.
*p*-value	0.798	0.651	0.439	
**FTD**				
rho	−0.041	−0.148	0.031	
95% CI	(−0.411 to 0.341)	−0.54 to 0.316	−0.388 to 0.439	n.a.
*p*-value	0.832	0.522	0.887	

Correlation coefficient (rho), 95% confidence interval (95% CI), and *p*-values from non-parametric spearman correlations are indicated

*AD* Alzheimer’s disease, *VaD* Vascular dementia, *LBD* Lewy body diseases, *CJD* Creutzfeldt-Jakob disease, *FTD* Fronto-temporal dementia, *t-Tau* CSF Total tau protein, *p-tau* CSF Phosphorylated tau protein, *Abeta42* CSF beta-amyloid 1-42, *Abeta40* CSF beta-amyloid 1-40

In a subset of *n* = 42 patients with AD, CSF LCN2 had previously been analyzed [13]. In paired samples, CSF and plasma LCN2 values showed a statistically significant positive correlation (rho = 0.318, 95% CI 0.007 to 0.574, *p* = 0.040) (Additional file 1D).

### Associations of plasma LCN2 with clinical and paraclinical data in AD

The relationship of plasma LCN2 concentrations and additional characteristics of the AD group were explored in subsets of patients with available data. The global cognitive status, evaluated by MMSE scores (*n* = 71 cases), showed no significant correlation with plasma LCN2, nor a clear tendency (rho = −0.009, 95% CI −0.248 to 0.232, *p* = 0.941) (Fig. 3B). The load of white matter hyperintensities as potential signs of concomitant cerebral small vessel disease was determined by the total score on the ARWMC scale (*n* = 54 cases) and showed no significant correlation with plasma LCN2 as well (rho = 0.002, 95% CI −0.273 to 0.277, *p* = 0.988) (Additional file 1E). In a comparison model including AD patients with the most frequent APOE genotypes (3/3, 3/4, and 4/4), no significant differences of LCN2 plasma levels between the three groups were observed (Fig. 3C). In all included AD patients, information of clinical disease progression was available. Plasma LCN2 concentrations were significantly lower (*p* = 0.013) in rpAD patients (mean: 49.1 ng/ml, SD ± 25.7) compared to spAD patients (mean: 63.3 ng/ml, SD ± 28.3) (Fig. 3D). Detailed results from the statistical models related to Fig. 3B–D are shown in Additional file 1C.

## Discussion

### Plasma Lipocalin 2 in the differential diagnosis of dementia

Our study revealed decreased plasma LCN2 in AD. The diagnostic accuracies versus control groups were moderate but interestingly, lower plasma LCN2 concentrations were statistically significant in comparison to all diagnostic groups including neurodegenerative (LBD, FTD, CJD), vascular (VD), and other (ND-Dem) causes for dementia syndromes. Only the MD group, which also included patients with AD-related (and vascular) pathology, showed similar mean values. The second important observation was the absence of significant associations between plasma LCN2 levels and CSF levels of other biomarkers of AD-related pathology.

Although, e.g., plasma p-tau has shown better diagnostic accuracy in other studies [5, 6], LCN2 may be a valuable additional marker reflecting a disease mechanism that is not directly associated with Abeta and tau pathology. Strikingly, the decrease of LCN2 seems to be disease specific. Other potential plasma markers of neurodegeneration (e.g., NfL and t-tau) or neuroinflammation (e.g., chitinase-3-like protein 1) were shown to be altered in AD but also in various other dementia syndromes [32, 33]. The potential as an alternative early biomarker, among newer biomarkers like fibrillary acidic protein (GFAP) [34] or soluble triggering receptor expressed on myeloid cells 2 (sTREM2) [35], is not clarified yet. Due to the case-control design of the study, the determination of the prognostic value of plasma LCN2 for AD patients was not possible but lowest concentrations were observed in rpAD patients, emphasizing the necessity of prospective studies on LCN2 in AD. Regarding other neurodegenerative diseases, no significant alterations compared to controls could be detected in LBDs, FTD, and sCJD. This suggests AD-specific rather than general neurodegenerative mechanisms leading to a decrease of plasma LCN2. On the other hand, data on plasma LCN2 in these diseases was not found in the literature and data on CSF LCN2 is scarce. Comparative neuropathological studies on LCN2 were only performed with AD and VaD brains [13].

Previous investigations found no significant differences of serum LCN2 levels between AD, MCI, and control groups [14, 18, 36] when matching or adjusting for age and sex as potential confounders. However, two of these studies showed significantly lower CSF LCN2 in MCI patients compared to controls [14, 36]. Regarding plasma LCN2, previous reports showed elevated levels in MCI patients [19] and in individuals with preclinical AD [20] compared to controls. Our investigation focused on healthy controls and differential diagnoses of dementia rather than prodromal or pre-clinical AD. MCI groups in this study were rather small and may not be appropriate to validate or refute previous results. Differences between AD and healthy controls were validated in an independent cohort and the differences compared to other dementias are consistent over the diagnostic groups. Unfortunately, the available data on LCN2 in AD and MCI, including our results, do not depict a coherent pattern and potential reasons are manifold. The usage of patients with subjective cognitive decline as controls [14, 18] has to be considered in the interpretation of results because those patients have an increased risk for the development of AD [37] and AD-related brain pathology may already be present at a pre-clinical stage [38]. Another factor may be the characterization of MCI. Studies that defined MCI by clinical syndrome criteria alone [19] might have included, at least in part, other than AD-related brain pathologies. Further, the evaluation and consideration of comorbidities differed between the studies. Time and condition of fluid sampling (e.g., after fasting or randomly throughout the day) may also play a role as LCN2 levels may be regulated by metabolic conditions and nutrients [39]. Finally, findings in serum and plasma may not be directly comparable [40]. All the aforementioned factors have to be considered in the design of future studies to guarantee a sound validation or refute of the current results.

### Associations of Lipocalin with biomarkers of AD-related pathology

A previous study reported significant positive association between CSF LCN2 and CSF Abeta42 in MCI patients [14]. An explanation might be that lower CSF LCN2 was associated with those patients with AD as underlying cause of MCI. In the AD group, the two biomarker levels did not significantly correlate, indicating that lower CSF LCN2 levels might be associated with the presence of AD-related pathology in a clinically defined MCI group rather than with the levels of Abeta42 in a homogenous AD group. In line with our results, the AD group showed no significant correlations between serum LCN2 and AD-related CSF biomarkers. On the other hand, this study and an earlier investigation reported significant inverse correlation of CSF Abeta42 with serum LCN2 in patients with subjective cognitive decline [14] and with plasma LCN2 in preclinical AD [20], respectively. The discrepancy of associations in plasma/serum and CSF at different stages of the disease has not been clarified and strongly suggests longitudinal analyzes in future studies.

### The pathophysiology of Lipocalin 2 in Alzheimer’s disease and vascular dementia

The pathophysiological background of plasma LCN2 and its alterations in body fluids of dementia patients is not deciphered, though. Neuropathological investigations from our study group revealed increased LCN2 immunoreactivity in macrophages and reactive astrocytes in the peripheral region of subacute infarcts and in the astrocytic scar [13] in VD. Experimental findings suggested that LCN2 is expressed in the brain during inflammatory response [41] to cerebral ischemia and hypoxia, and mediates additional brain damage and cognitive decline in VD [12, 42, 43]. In the present study, we observed statistically significant elevation of LCN2 in plasma of VD patients compared to AD patients but only a non-significant tendency towards higher values compared to controls, diminishing the potential of high plasma LCN2 as a biomarker for VD. In contrast to our previous observations in the CSF of VD patients [13], plasma LCN2 levels were not associated with the degree of white matter hyperintensities in AD. This might be explained by the low overall frequency of white matter hyperintensities (compared to VD) or the possibility that some of these lesions are rather associated with neurodegeneration than with cerebral small vessel disease in AD [44].

Regarding the pathophysiology of LCN2 in AD, only few experimental and neuropathological data are available. Similar to VD, increased LCN2 immunoreactivity was observed in reactive astrocytes in AD brain samples [13] and cell models showed that LCN2 modulates Abeta toxicity in astrocytes [45]. On the other hand, LCN2 deficiency in an AD mouse model led to decreased iron accumulation in the hippocampus but not to altered symptoms, amyloid plaque load, or glial activation [46]. Interestingly, peripheral LCN2 was shown to be associated with executive dysfunction in preclinical and mild AD patients rather than with memory impairment [20, 47], which is the typical early symptom of AD-related pathology. This suggests that LCN2 might possibly reflect secondary mechanisms in the complex pathophysiology of AD. Results from our group indicate that CSF LCN2 levels are similar in VD and MD [13] but in contrast, this study shows that plasma LCN2 levels may be similar in AD and MD. Whereas high CSF LCN2 levels in VD may be explained by increased expression in the brain as part of an inflammatory response to hypoxic-ischemic injury, the reasons for low peripheral levels in AD, MD, and MCI patients remain obscure. The question whether LCN2 in the brain is “friend or foe” [48] is not fully answered, yet.

### Study strengths and limitations

The strength of this study is the broad selection of clinical relevant differential diagnosis as well as healthy individuals as control groups. Patients were characterized by a thorough clinical work-up, offering the opportunity to explore relations of plasma LCN2 in AD. On the other hand, the number of patients in each group, especially in the validation cohort and in MCI groups, was rather low. Some weak differences or relationships may not have become apparent in statistical analyzes. Structured prospective follow-up data and biological samples were not available.

Plasma LCN2 was decreased in AD compared to all other groups in this study but only part of plasma samples from AD and other patients were paired with available CSF samples. We could not perform evaluation of plasma and CSF LCN2 in parallel. In our previous investigation, the AD group showed the lowest mean CSF LCN2 level among the diagnostic groups but in the multi-comparison model, statistical significance could only be detected for elevated levels in VaD [13]. The different findings in CSF and plasma may be caused by different sample sizes, applied statistical models, or patient characteristics but need to be investigated in larger investigations with paired samples.

Further, the study is of an exploratory nature. Associations of biomarkers were assessed through non-parametric Spearman correlations and confounders were not included in this statistical model. Comorbidities that might influence LCN2 levels were only excluded in HC, MCI, and AD groups but may potentially be present in other dementia groups. Future investigations will have to consider potential demographic confounders and comorbidities, as well as other factors such as time of blood sampling at the stage of designing the study.

## Conclusions

In conclusion, plasma LCN2 is a promising additional biomarker for the diagnosis of AD. In our opinion, the most salient and striking results are the specific decrease in AD and the lack of correlation with Abeta and p-tau levels. LCN2 seems to be relatively independent from other markers of neuro-axonal injury and AD-related pathology, offering high potential value as part of a diagnostic composite biomarker or as a surrogate marker in clinical interventions that aim specific disease mechanisms in AD, e.g., neuro-inflammation. Still, the pathophysiological background of LCN2 in AD as well as the discrepant findings in different stages of the disease has to be explored further.

## Supplementary Information

**Additional file 1: A.** Pre-analytic study. Four plasma samples (healthy controls) were analyzed repeatedly at baseline and each time after three transfers (upper left), three freeze-thaw cycles (room temperature/minus 80°C, upper right), one to four and eight days storage at 4°C (lower left), as well as room temperature (lower right). Comparisons of Lipocalin 2 concentrations were calculated with ANOVA followed by Bonferroni correcture. Differences are indicated when *p* was < 0.05 (*) and < 0.001 (***), respectively. **B.** Results from linear regression models and post hoc Tests in Fig. 1. Estimates, standard errors, t-values, and *p*-values were calculated through pairwise comparisons of means of log-transferred values by Tukey contrasts, HC: healthy controls, ND-Dem: non-neurodegenerative neurological diseases with dementia syndrome, AD: Alzheimer’s disease, CJD: Creutzfeldt-Jakob disease, LBD: Lewy body diseases (dementia with Lewy bodies and Parkinson’s disease dementia), FTD: fronto-temporal dementia, and VaD: vascular dementia. **C.** Results from linear regression models and post hoc Tests in Figs. 2 and 3. Estimates, standard errors, t-values, and *p*-values were calculated through pairwise comparisons of means of log-transferred values by Tukey contrasts, HC: healthy controls, AD: Alzheimer’s disease, MCI-AD: mild cognitive impairment with positive AD biomarker, VaD: vascular dementia, VCI-MCI: mild vascular cognitive impairment, spAD: slowly progressive AD; rpAD: rapidly progressive AD. **D.** Results from linear regression models using only A+/T+ AD-patients. Estimates, standard errors, t-values, and *p*-values were calculated through pairwise comparisons of means of log-transferred values by Tukey contrasts, HC: healthy controls, ND-Dem: non-neurodegenerative neurological diseases with dementia syndrome, AD: Only patients with diagnosis of Alzheimer’s disease based on pathologic CSF abeta 1-42 and also pathologic phosphorylated tau protein (A+/T+, *n* = 39), CJD: Creutzfeldt-Jakob disease, LBD: Lewy body diseases (dementia with Lewy bodies and Parkinson’s disease dementia), FTD: fronto-temporal dementia, and VaD: vascular dementia. **E.** Correlation of plasma and CSF LCN2. Association of plasma and CSF lipocalin 2 (LCN2) in paired samples from the AD group in the study cohort. Correlation coefficient (rho), 95% confidence interval (95% CI) and *p*-values from non-parametric spearman correlation is indicated. **F.** Correlation of plasma LCN2 and ARWMC in AD. Scatter plot of the association between age-related white matter changes scale (ARWMC) scores and plasma lipocalin LCN2 concentrations. Spearman coefficients (cc) with 95% confidence interval (CI) and corresponding *p*-values are indicated.

## Data Availability

The datasets used and analyzed during the current study are available from the corresponding author on reasonable request.

## Abbreviations

AD: Alzheimer’s disease
CSF: Cerebrospinal fluid
p-tau: Phosphorylated Tau protein
Abeta42: Beta-amyloid 1-42
t-tau: Total-Tau
PET: Positron emission tomography
NfL: Neurofilament light chain
LCN2: Lipocalin 2
VaD: Vascular dementia
MCI: Mild cognitive impairment
HC: Healthy controls
CJD: Creutzfeldt-Jakob disease
ARWMC: Age-related white matter changes
MMSE: Mini Mental Status Examination
spAD: Slowly progressive AD
rpAD: Rapidly progressive AD
MCI-VCI: Mild vascular cognitive impairment
MD: Mixed dementia
LBD: Lewy bodies and Parkinson’s disease dementia
FTD: Fronto-temporal dementia
ND-Dem: Neurological diseases and dementia of primarily non-neurodegenerative causes
ROC: Receiver operating characteristic
AUC: Area under the curve
