# Supplementary material for: Plasma Lipocalin 2 in Alzheimer’s disease: potential utility in the differential diagnosis and relationship with other biomarkers
Source: Alzheimers Res Ther. 2022 Jan 13;14:9. doi: 10.1186/s13195-021-00955-9 (PMC8759265; doi:10.1186/s13195-021-00955-9)
Supplement: Supplementary file 1 — Additional file 1: A. Pre-analytic study. Four plasma samples (healthy controls) were analyzed repeatedly at baseline and each time after three transfers (upper left), three freeze-thaw cycles (room temperature/minus 80°C, upper right), one to four and eight days storage at 4°C (lower left), as well as room temperature (lower right). Comparisons of Lipocalin 2 concentrations were calculated with ANOVA followed by Bonferroni correcture. Differences are indicated when p was < 0.05 (*) and < 0.001 (***), respectively. B. Results from linear regression models and post hoc Tests in Fig. 1. Estimates, standard errors, t-values, and p-values were calculated through pairwise comparisons of means of log-transferred values by Tukey contrasts, HC: healthy controls, ND-Dem: non-neurodegenerative neurological diseases with dementia syndrome, AD: Alzheimer’s disease, CJD: Creutzfeldt-Jakob disease, LBD: Lewy body diseases (dementia with Lewy bodies and Parkinson’s disease dementia), FTD: fronto-temporal dementia, and VaD: vascular dementia. C. Results from linear regression models and post hoc Tests in Figs. 2 and 3. Estimates, standard errors, t-values, and p-values were calculated through pairwise comparisons of means of log-transferred values by Tukey contrasts, HC: healthy controls, AD: Alzheimer’s disease, MCI-AD: mild cognitive impairment with positive AD biomarker, VaD: vascular dementia, VCI-MCI: mild vascular cognitive impairment, spAD: slowly progressive AD; rpAD: rapidly progressive AD. D. Results from linear regression models using only A+/T+ AD-patients. Estimates, standard errors, t-values, and p-values were calculated through pairwise comparisons of means of log-transferred values by Tukey contrasts, HC: healthy controls, ND-Dem: non-neurodegenerative neurological diseases with dementia syndrome, AD: Only patients with diagnosis of Alzheimer’s disease based on pathologic CSF abeta 1-42 and also pathologic phosphorylated tau protein (A+/T+, n = 39), CJD: Creutzfeldt [file 13195_2021_955_MOESM1_ESM.pdf]

Additional file 1 A. Pre-analytic study

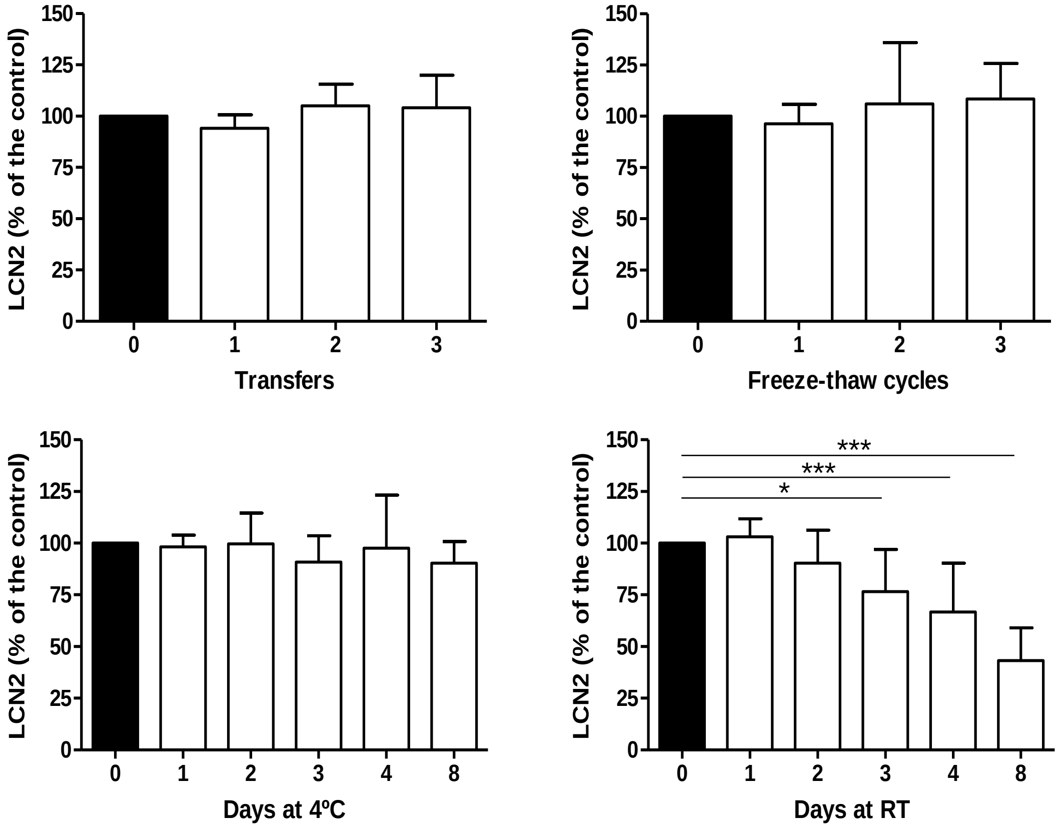

Figure Legend:

Four plasma samples (healthy controls) were analyzed repeatedly at baseline and each time after three transfers (upper left), three freeze-thaw cycles (room temperature/minus 80°C, upper right), one to four and eight days storage at 4°C (lower left), as well as room tempreature (lower right). Comparisons of Lipocalin 2 concentrations were calculated with ANOVA followed by Bonferroni correcture. Differences are indictated when p was < 0.05 (*) and < 0.001 (***), respectively.

Additonal file 1 B. Results from linear regression models and post hoc Tests in Figure 1.

Comparison	Estimate	Std. Error	t-value	p-value
CJD - AD	0.552	0.082	6.771	<0.001
FTD - AD	0.404	0.110	3.659	0.005
HC - AD	0.600	0.082	7.286	<0.001
LBD - AD	0.369	0.097	3.811	0.003
ND-Dem - AD	0.422	0.120	3.527	0.008
VaD - AD	0.666	0.114	5.849	<0.001
FTD - CJD	-0.148	0.108	-1.367	0.811
HC - CJD	0.048	0.080	0.594	0.997
LBD - CJD	-0.183	0.096	-1.904	0.467
ND-Dem - CJD	-0.130	0.117	-1.109	0.921
VaD - CJD	0.114	0.112	1.014	0.948
HC - FTD	0.196	0.109	1.793	0.542
LBD - FTD	-0.035	0.121	-0.285	1
ND-Dem - FTD	0.018	0.139	0.133	1
VaD - FTD	0.262	0.135	1.942	0.441
LBD - HC	-0.230	0.096	-2.412	0.189
ND-Dem - HC	-0.177	0.118	-1.502	0.734
VaD - HC	0.066	0.114	0.576	0.997
ND-Dem - LBD	0.053	0.131	0.405	1
VaD - LBD	0.296	0.124	2.392	0.197
VaD - ND-Dem	0.243	0.143	1.700	0.605

Estimates, standard errors, t-values, and p-values were calculated through pairwise comparisons of means of log-transferred values by Tukey contrasts, HC: healthy controls, ND-Dem: non-neurodegenerative neurological diseases with dementia syndrome, AD: Alzheimer's disease, CJD: Creutzfeldt-Jakob disease, LBD: Lewy body diseases (dementia with Lewy bodies and Parkinson's disease dementia), FTD: fronto-temporal dementia, and VaD: vascular dementia.

Additonal file 1 C. Results from linear regression models and post hoc Tests in Figure 2 and Figure 3.

Comparison	Estimate	Std. Error	t-value	p-value
**Figure 2A**				
HC - AD	0.580	0.083	6.946	<0.001
MCI-AD - AD	0.590	0.1467	4.012	<0.001
MCI-AD - HC	0.009	0.1476	0.063	0.998
**Figure 2B**				
HC - AD	0.355	0.112	3.164	0.007
amnestic MCI - AD	0.248	0.106	2.350	0.056
MCI-AD - HC	-0.107	0.106	-1.011	0.572
**Figure 2D**				
HC - VaD	0.165	0.137	1.204	0.451
VCI-MCI - HC	-0.001	0.158	-0.008	1.000
VCI-MCI -VaD	-0.166	0.169	-0.982	0.587
**Figure 3**				
spAD - rpAD	0.314	0.124	2.539	0.013
Apo E 3/4 - 3/3	0.063	0.147	0.435	0.899
Apo E 4/4 - 3/3	0.337	0.239	1.409	0.336
Apo E 4/4 - 3/4	0.273	0.235	1.164	0.471
A+T-N+/- vs. A-T+N+/-	0.199	0.212	0.941	0.768
A+T+N+/- vs. A-T+N+/-	0.068	0.159	0.428	0.971
A+T+N+/- vs. A+T-N+/-	-0.131	0.189	-0.694	0.891
A-T+N+/- vs. A-T-N+	0.063	0.419	0.150	0.999
A+T-N+/- vs. A-T-N+	0.262	0.419	0.625	0.917
A+T+N+/- vs. A-T-N+	0.131	0.406	0.322	0.987

Estimates, standard errors, t-values, and p-values were calculated through pairwise comparisons of means of log-transferred values by Tukey contrasts, HC: healthy controls, AD: Alzheimer's disease, MCI-AD: mild congnitive impairment with positive AD biomarker, VaD: vascular dementia, VCI-MCI: mild vascular cognitivie impairment

Additional file 1 D. Results from linear regression models using only A+/T+ AD-patients.

Comparison	Estimate	Std. Error	t-value	p-value
CJD - AD	0.551	0.099	5.562	<0.001
FTD - AD	0.403	0.124	3.246	0.021
HC - AD	0.600	0.101	5.954	<0.001
LBD - AD	0.370	0.114	3.250	0.021
ND-Dem - AD	0.421	0.132	3.189	0.025
VaD - AD	0.665	0.128	5.210	<0.001

Estimates, standard errors, t-values, and p-values were calculated through pairwise comparisons of means of log-transferred values by Tukey contrasts, HC: healthy controls, ND-Dem: non-neurodegenerative neurological diseases with dementia syndrome, AD: Only patients with diagnosis of Alzheimer's disease based on pathologic CSF abeta 1-42 and also pathologic phosphorylated tau protein (A+/T+, n=39), CJD: Creutzfeldt-Jakob disease, LBD: Lewy body diseases (dementia with Lewy bodies and Parkinson's disease dementia), FTD: fronto-temporal dementia, and VaD: vascular dementia.

Additional file 1 E. Correlation of plasma and CSF LCN2

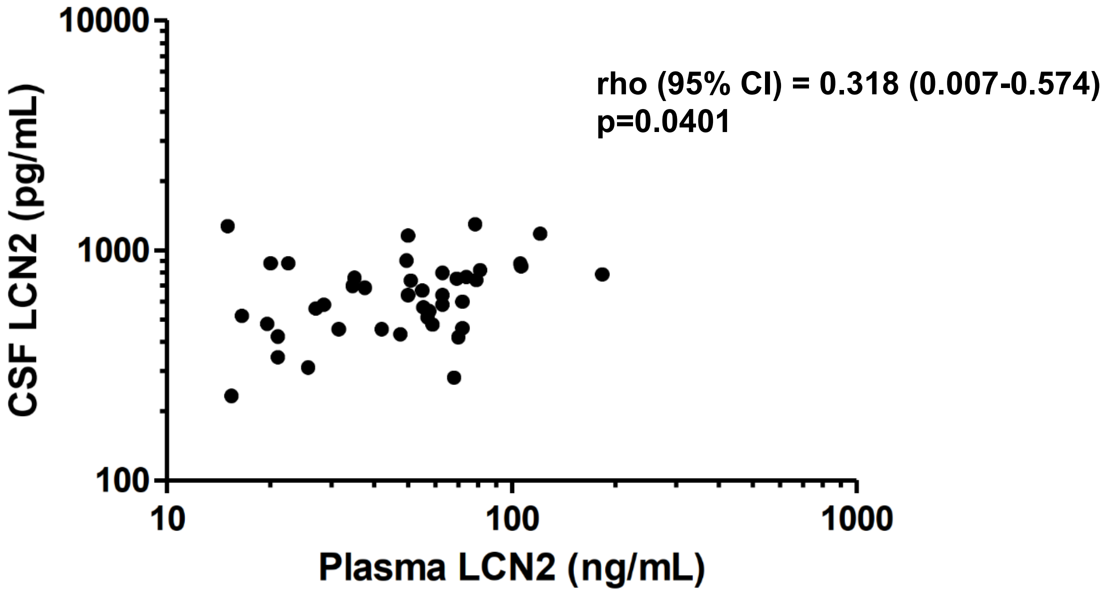

Figure legend:

Association of plasma and CSF lipocalin 2 (LCN2). Correlation coefficient (rho), 95% confidence interval (95% CI) and p-values from non-parametric spearman correlation is indicated.

Additional file 1 F. Correlations of plasma LCN2 and ARWMC in AD

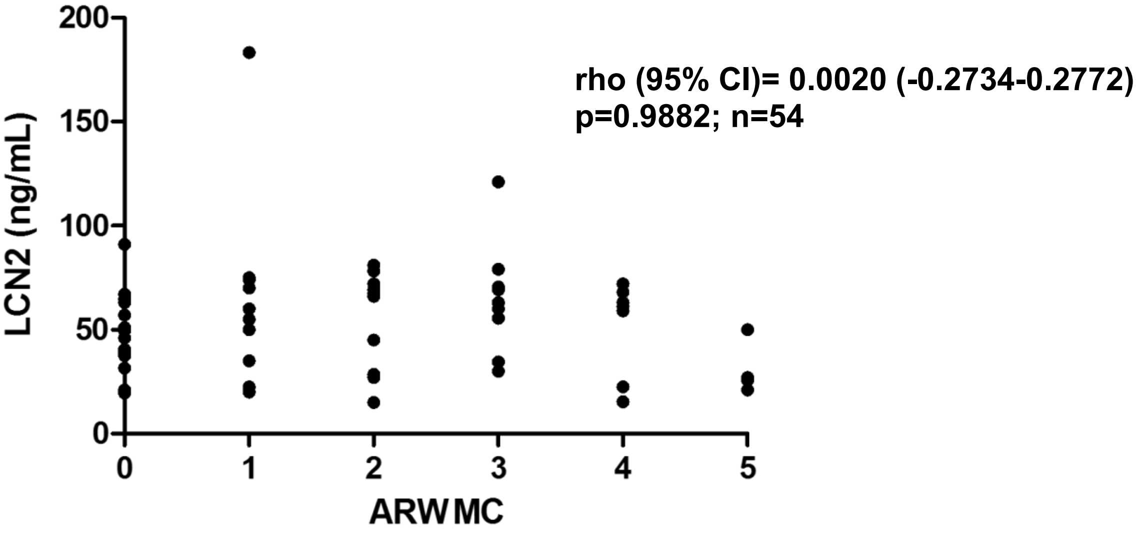

Figure legend:

Scatter plot of the association between age-related white matter changes scale (ARWMC) scores and plasma lipocalin LCN2 concentrations. Spearman coefficients (cc) with 95% cinfidence interveal (CI) and corresponding p-values are indicated.
